# Malaria: a reemerging disease in Africa.

**DOI:** 10.3201/eid0403.980313

**Published:** 1998

**Authors:** T C Nchinda

**Affiliations:** World Health Organization, Geneva, Switzerland. nchindat@who.ch

## Abstract

A recent upsurge of malaria in endemic-disease areas with explosive epidemics in many parts of Africa is probably caused by many factors, including rapidly spreading resistance to antimalarial drugs, climatic changes, and population movements. In Africa, malaria is caused by Plasmodium falciparum and is transmitted by Anopheles gambiae complex. Control efforts have been piecemeal and not coordinated. Strategies for control should have a solid research base both for developing antimalarial drugs and vaccines and for better understanding the pathogenesis, vector dynamics, epidemiology, and socioeconomic aspects of the disease. An international collaborative approach is needed to build appropriate research in a national context and to effectively translate research results into practical applications in the field. The Multilateral Initiative for Malaria in Africa can combine all of the above strategies to plan and coordinate partnerships, networking, and innovative approaches between African scientists and their Northern partners.


### Malaria: A Reemerging Disease in Africa

**Thomas C. Nchinda**
World Health Organization, Geneva, Switzerland

A recent upsurge of malaria in endemic-disease areas with explosive epidemics in many parts of Africa is probably caused by many factors, including rapidly spreading resistance to antimalarial drugs, climatic changes, and population movements. In Africa, malaria is caused by *Plasmodium falciparum* and is transmitted by *Anopheles gambiae* complex. Control efforts have been piecemeal and not coordinated. Strategies for control should have a solid research base both for developing antimalarial drugs and vaccines and for better understanding the pathogenesis, vector dynamics, epidemiology, and socioeconomic aspects of the disease. An international collaborative approach is needed to build appropriate research in a national context and to effectively translate research results into practical applications in the field. The Multilateral Initiative for Malaria in Africa can combine all of the above strategies to plan and coordinate partnerships, networking, and innovative approaches between African scientists and their Northern partners.

The global malaria eradication program of the 1950s and 1960s suffered serious setbacks in the early 1970s, and the disease was slowly increasing in areas of Asia and South America where the number of cases had been reduced to low levels. This article discusses malaria and, more specifically, malaria in Africa, where the global eradication program was never started and the disease is reemerging at an alarming and unprecedented rate.

### The Disease

Malaria in humans is caused by a protozoon of the genus *Plasmodium* and the four subspecies, *falciparum, vivax, malariae*, and *ovale*. The species that causes the greatest illness and death in Africa is *P. falciparum*. The disease is transmitted by the bites of mosquitoes of the genus *Anopheles,* of which the *Anopheles gambiae* complex (the most efficient) is responsible for the transmission of disease in Africa. Fever is the main symptom of malaria. The most severe manifestations are cerebral malaria (mainly in children and persons without previous immunity), anemia (mainly in children and pregnant women), and kidney and other organ dysfunction (e.g., respiratory distress syndrome). Persons repeatedly exposed to the disease acquire a considerable degree of clinical immunity, which is unstable and disappears after a year away from the endemic-disease environment. Immunity reappears after malarial bouts if the person returns to an endemic-disease zone. Most likely to die of malaria are persons without previous immunity, primarily children or persons from parts of the same country (e.g., high altitudes) where transmission is absent, or persons from more industrialized countries where the disease does not exist.

Address for correspondence: Thomas C. Nchinda, Global Forum for Health Research, World Health Organization, 1211 Geneva 27, Switzerland; fax: 41-22-791-4394; e-mail: nchindat@who.ch.

### Why Is Malaria Reemerging?

In the last decade, the prevalence of malaria has been escalating at an alarming rate, especially in Africa. An estimated 300 to 500 million cases each year cause 1.5 to 2.7 million deaths, more than 90% in children under 5 years of age in Africa (1). Malaria has been estimated to cause 2.3% of global disease and 9% of disease in Africa (1); it ranks third among major infectious disease threats in Africa after pneumococcal acute respiratory infections (3.5%) and tuberculosis (TB) (2.8%). Cases in Africa account for approximately 90% of malaria cases in the world (1). Between 1994 and 1996, malaria epidemics in 14 countries of sub-Saharan Africa caused an unacceptably high number of deaths, many in areas previously free of the disease (2).

Adolescents and young adults are now dying of severe forms of the disease. Air travel has brought the threat of the disease to the doorsteps of industrialized countries, with an increasing incidence of imported cases and deaths from malaria by visitors to endemic-disease regions. The estimated annual direct and indirect costs of malaria were US$800 million in 1987 and were expected to exceed US$1.8 billion by 1995 (3).

A number of factors appear to be contributing to the resurgence of malaria: 1) rapid spread of resistance of malaria parasites to chloroquine and the other quinolines; 2) frequent armed conflicts and civil unrest in many countries, forcing large populations to settle under difficult conditions, sometimes in areas of high malaria transmission; 3) migration (for reasons of agriculture, commerce, and trade) of nonimmune populations from nonmalarious and usually high to low parts of the same country where transmission is high; 4) changing rainfall patterns as well as water development projects such as dams and irrigation schemes, which create new mosquito breeding sites; 5) adverse socioeconomic conditions leading to a much reduced health budget and gross inadequacy of funds for drugs; 6) high birth rates leading to a rapid increase in the susceptible population under 5 years of age; and 7) changes in the behavior of the vectors, particularly in biting habits, from indoor to outdoor biters.

### What Knowledge Is Needed for Effective Control?

Continental sub-Saharan Africa was never a part of the global malaria eradication program. The severity of the disease, the density and efficiency of *An. gambiae*, the problem of eradicating the disease over such a large land mass with recurrent reinvasions, high costs, and subsequent maintenance must have all contributed to the lack of will to undertake an eradication program. Also, the eradication program period coincided with the colonial and immediate postcolonial period, during which little or no indigenous capacity was available to initiate and sustain malaria eradication. After a period of laissez faire regarding malaria control, these countries have had to face the reemergence of the disease. Important questions about control include the following. Is there enough knowledge about the disease and its determinants? Are there enough tools? Are existing resources adequate? Are governments and populations of endemic-disease countries adequately prepared?

### Knowledge About the Disease and Its Determinants

Falciparum malaria is a complex disease with a patchy nonuniform distribution and clinical manifestations that vary from one area to another within an endemic-disease zone, often showing space-time clustering of severe malaria in the community (4). The relationship between fevers, clinical disease, anemia, and cerebral malaria remains the subject of current research. The determinants of severe life-threatening malaria need further elucidation. Present research, focusing on the disease rather than the infection and the dynamics of its transmission, is bringing in new vision about the disease, particularly the immunologic aspects. Persons with asymptomatic parasitemia constitute an important reservoir. The epidemiology of malaria (particularly the relationship between the clinical patterns of the disease in different locations, the pattern of severe disease, and causes of deaths due to malaria) needs future research (5).

### Tools for Malaria Control

The present strategy for malaria control, adopted by the Ministerial Conference on Malaria in Amsterdam in 1992, is to prevent death, reduce illness, and decrease social and economic loss due to the disease (6). Its practical implementation requires two main tools: first, drugs for early treatment of the disease, management of severe and complicated cases, and prophylactic use on the most vulnerable population (particularly pregnant women); second, insecticide-treated nets for protection against mosquito bites. Each tool has its own problems in regard to field implementation.

Chloroquine remains the first-line therapy for malaria. However, the alarming increase in resistance in eastern and southern Africa requires that sulfadoxine-pyrimethamine replace chloroquine as the first-line drug. Currently, 20% to 30% of strains are highly resistant (RIII) with in vivo levels of 40% to 60%. Resistance has been spreading westward, attaining levels of 20% to 35% in West Africa. Chloroquine remains the drug of choice in most of sub-Saharan Africa.

Resistance to mefloquine, another first-line drug, developed in the early 1980s, was noticed soon after its introduction and is now almost at the same level as chloroquine. Sulfadoxine-pyrimethamine (Fansidar, Hoffman la Roche) is the second-line drug in many countries of West and Central Africa, but so much resistance appears to be rising in countries of East Africa that atovaquone/dapsone (Malarone, Glaxo Wellcome) is being developed as a replacement. Intravenous quinine is still the main therapy for cerebral malaria, although resistance is increasing. Development by the African strains of malaria parasites of the pattern of drug resistance now seen in Southeast Asia would be a major disaster.

More research is needed. For example, it is necessary to initiate systematic monitoring of drug resistance in Africa using standardized methods. Drug efficacy studies using in vivo methods have now been standardized by the World Health Organization (WHO)/Regional Office for Africa (AFRO) and carried out in a large number of countries in West, Central, and East Africa. Sentinel sites have also been established for monitoring resistance. No new methods are being developed. The feasibility of using polymerase chain reaction techniques should be explored. Also, management guidelines should be developed concerning when and under what conditions to change the treatment regimen for different levels of resistance at the district, regional, and central level. Development and field testing of inexpensive, effective new malaria drugs are urgently needed to replace present drugs when resistance patterns make them unusable. Drugs developed because of the more serious problem of drug resistance in Asia should be field tested in Africa. The most promising ones, artemisinin and its derivatives artemether, arteether, and artesunate, are being tested for use in cerebral malaria and cases of proven resistance to chloroquine (12); some are already used in some countries.

Research carried out in Dakar (7) demonstrated the efficacy of insecticide-treated nets for reducing infant death; subsequent large-scale multicenter studies in six countries across Africa confirmed this finding (8-10). However, costs of the nets and treatment still inhibit wide-scale use. Ongoing research seeks ways of reducing these costs, such as social marketing, possible involvement of the private sector, cost-effective methods for net treatment, the most appropriate nets, and proper procurement of insecticides and treatment of the nets. Eventually, the long-term effects on natural acquisition of partial immunity to malaria in endemic-disease areas should be evaluated. The old vector-control method of house spraying persists in some countries. The relative merits and cost-effectiveness of house spraying versus the use of treated nets should be evaluated.

### The Challenge of Malaria Control to Communities and Governments

The best tools will not necessarily lead to malaria control. African populations have traditional perceptions about disease causation and management. Some diseases are considered suitable for management by western medicine, while others are considered the exclusive domain of local traditional health practitioners. Decisions to seek western medicine for any illness are often considered a last resort. Studies on health-seeking behavior, perceptions of malaria, treatments, and decision making for health care at the household level are crucial to malaria control. Such studies must be accompanied by improved public awareness of the importance of seeking appropriate treatment and complying with recommended regimens.

Management of disease in the household devolves on mothers. Fever remains the most recognized symptom of malaria. Studies are ongoing to determine the proportion of fevers actually due to malaria. Mothers should be taught to recognize the symptoms of malaria, to provide home management, and to know when to refer cases to health centers. Four countries in Africa have developed and tested teaching guides to facilitate home management of malaria (11). Also, guidelines for the management of fever at the periphery have been developed and field tested within the Sick Child Initiative and have been recommended for wide-scale application. Socioeconomic and community studies are needed to understand the extent to which the communities will participate in new malaria control measures. Finally, cost recovery of health care, including costs of drugs (the Bamako Initiative), has been the subject of many recent studies and probably holds the key to health care in rural populations.

Some study results indicate an initial fall in use of services following the introduction of cost-recovery schemes (12). However, a recent study indicates the opposite. Community health workers were trained to administer prepackaged antimalarial drugs only when paid. They also received direct remuneration for their work rather than being supported by the village on a voluntary basis (13). This plan seems to have increased attendance. This subject needs large-scale multicenter studies.

### Governments' Response—Peripheral Health Services

Health service organization, function, and governing policies are important to malaria control. Health policy and systems research have been recently identified as neglected areas of research in need of international effort (1). Many studies are researching different ways to integrate vertical malaria control programs into the general health-care system. Economic evaluation of different interventions is important, and the techniques are continually being refined and improved. They require much local capacity since they tend to be country specific. Studies in this area have now caught up with the current trend favoring decentralization of services, giving more power to the districts. Such studies include ways of improving case management where health services have been decentralized, sustaining effective interventions, and ensuring that drug supply chains function optimally. Extensive research is examining health sector reform on malaria control (12). Health sector reform holds great potential for controlling malaria and all other diseases, as it is the focal point of the central and local governments and the populations themselves. Other needed research includes different health policies, access to health services, and the issues of equity in health care.

### Is There a Place for Biomedical Research?

If the emphasis appears to be on epidemiologic and socioeconomic research and studies on health policies and systems, it is because these results have immediate importance to malaria control. The argument is for better use of existing tools. However, tools alone will not provide all the knowledge needed for sustainable malaria control. Recent research by the Wellcome Trust and the National Institutes of Health on sequencing the genome of *P. falciparum* is likely to lead to development of new antimalarial drugs and vaccines. Similarly, DNA technologies are being used to search for candidate molecules for vaccines and new targets for drug development.

The development of a malaria vaccine is still in the laboratories, and no effective vaccine is in sight despite promising candidates. Subsequently, all candidate vaccine trials must be closely linked to studies on how humans acquire immunity and the correlation between protective immunity and immunologic assays. Such studies should be carried out longitudinally in multiple sites where future vaccines will be tested.

On the vector side, studies in Mali have shown that malaria transmission in this Sahel country is maintained by a relay transmission pattern, whereby the three main vectors appear at different times of the year, thus ensuring that vectors are always present (Y. Toure, pers. comm.). More research is in progress concerning the potential of using genetic engineering to make the main malaria vector, *An. gambiae*, refractory to the malaria parasite and releasing this refractory parasite into the wild population to replace the active vectors. Despite potential ethical problems, this approach probably constitutes a long-term future method for interrupting malaria transmission (14). Finally, the much-neglected issue of the pathogenesis of malaria anemia both in children and pregnant women, as well as the link of anemia in pregnancy and HIV/AIDS, needs further study and is likely to be multifactorial.

Mapping malaria transmission intensity using geographic information systems and geographic positioning systems has developed into a Pan-African research collaboration for Mapping the Malaria Risk in Africa, which has received international funding. It plays a major role in time-spatial mapping of malaria across the continent with a strong potential for predicting malaria epidemics (15) and monitoring control.

### What Is the Response of the World Health Organization?

WHO developed global and regional strategies for malaria control after the Ministerial Conference on Malaria in Amsterdam in 1992. WHO/AFRO has multiplied efforts to encourage countries to embark seriously on malaria control. A WHO/AFRO Task Force for Malaria comprising a selected sample of malaria control managers, malaria experts from Africa, and technical representatives from bilateral and multilateral agencies funding malaria control in Africa was set up in 1994. This task force has met regularly to provide guidance on malaria control strategies and to recommend criteria for monitoring and evaluation as well as operational research. Some of these agencies have recently increased their malaria control funding directly to some countries of Africa; others have preferred funding through the regional office.

In addition, the WHO Director General made a generous grant of US$10 million from the WHO regular budget for 1997 for intensified malaria control efforts. Momentum is building, strongly supported by the World Bank, for more concerted efforts at malaria control.

### The Way Forward

The Multilateral Initiative on Malaria in Africa (MIM) was created in Dakar in January 1997 from the realization that success in controlling malaria in the future would be greatly enhanced by cooperation and collaborative efforts in research to support strategies for control (5). MIM capitalized on the important 1992 Ministerial Conference, which led to the adoption of a Global Plan of Action for Malaria Control and the World Health Assembly Resolution on this subject (WHA 49.11), urging increased efforts on malaria control. Composed of scientists from Africa and their colleagues from industrialized countries as well as representatives from major funding agencies, MIM plans to facilitate collaboration between governments, research scientists, research funding agencies, and the private (pharmaceutical industry) sector for concerted action through research to combat malaria.

Like other diseases of low-income countries, malaria has been grossly underfunded. From 1990 to 1992, $58 million a year was spent on malaria research, while $56 billion was spent on health research worldwide. Expressed as research investment per death, malaria research receives about US$42 per fatal case, much less than for other diseases such as HIV/AIDS (US$3,270) and asthma (US$789) (3). Rather than the duplicative efforts of the past, MIM encourages a common goal with common research priorities, which should create a greater spirit of cooperation.

### Strengthening Research Capability

MIM took a firm stand on indigenous capacity building for malaria research in Africa, an important prerequisite for sustainable research and control of malaria in that continent. Training would be carried out in Africa as far as possible but not exclusively so. Training would be carried out for all health-care workers within the malaria research and control pyramid, including Ministry of Health personnel and those in research institutes and universities, with no exclusion. Flexible training programs would be developed to meet the needs of individual research centers and countries. A good start has been made. Using funds provided late in 1997 to WHO's Tropical Diseases Research Programme, a task force was set up for Malaria Research Capability Strengthening in Africa. The money funded North/South and South/South collaborative research in malaria. All the principal investigators were to be from Africa. Training was central to the projects so that more hands-on and practical research training would be given to trainees, and practical refresher training and technology transfer would be given to experienced scientists.

Research centers also need to be strengthened. Laboratories need refurbishing and equipment and supplies (including computer equipment and software), and vehicles are needed for field studies. Suitable research careers should be created to encourage the best scientists to remain in research.

Because scientific isolation constitutes a major constraint to African scientists, communication facilities need urgent attention. One of MIM's highest priorities is to enhance the capacity of African scientists to communicate electronically with each other and with colleagues around the world and to access needed scientific information from local and remote libraries and the Internet. NIH's National Library of Medicine is playing a lead role in this critical area.

### The Future

Malaria is an important social, economic, and developmental problem affecting individuals, families, communities, and countries. The best chance for successfully combating the disease requires a collaboration particularly of those responsible for control and research. Such collaboration, particularly between South and North, is being actively developed, and MIM presents itself as a worthwhile initiative (16). Important factors are 1) placing the control strategy on a strong research base, 2) strong international collaboration, and 3) sustained government support.

Smallpox was eradicated because of the development of freeze-dried vaccine, the development of the multiple-use nozzle jet injector and bifurcated needle, and the replacement of mass vaccination by selective vaccination, coupled with a strong international effort. Onchocerciasis is being controlled because research results were immediately applied to control. Translating research findings into control methods has also been pursued for Chagas disease and leprosy. Concerted action between the research and control communities is needed to ensure that malaria follows the same path. MIM strongly advocates this approach. Research must be a constant feature throughout the entire process of malaria control.

Dr. Nchinda is senior health specialist at the Global Forum for Health Research based at the World Health Organization headquarters in Geneva. His research interests are in tropical diseases research and control, particularly malaria, training and utilization of health personnel, research capacity strengthening, health services research, and organization of community health services.

### References

1. World Health Organization. Investing in health research for development. Report of the Ad Hoc Committee on Health Research Relating to Future Intervention Options. Geneva:The Organization; 1996. Report No.: TDR/Gen/96.1
2. Harare declaration on malaria prevention and control in the context of African economic recovery and development. In: Proceeding of the 33rd Ordinary Session of the Assembly of Heads of State and Government, Organization of African Unity; 1997 2-4 June; Harare, Zimbabwe.
3. Anderson J, MacLean M, Davies C. Malaria research: an audit of international activity. Prism Report No. 7, The Wellcome Trust; 1996.
4. Snow RW, Schellenberg JR, Peshu N, Foster D, Newton CR, Witstanley PA, et al. Periodicity and space-time clustering of severe childhood malaria on the coast of Kenya. Trans R Soc Trop Med Hyg 1993;87:386-90.
5. Final report: International Conference on Malaria in Africa, 6-9 January 1997, Dakar, Senegal. [document online] Available from: url: http://www.niaid.nih/dmid/malafr/.
6. World Health Organization. Control of Tropical Diseases: 1. Progress Report. Geneva: The Organization, Division of Control of Tropical Diseases; 1994. Report No.: CTD/MIP/94.4
7. Alonso PL, Lindsay SW, Armstrong JRM, Conteh M, Hill AG, David PH, et al. The effect of insecticide-treated bed nets on mortality of Gambian children. Lancet 1991;337:1499-502.
8. Nevill CG, Some ES, Mung'ala VO, Mutemi W, New L, Marsh K, et al. Insecticide treated bednets reduce mortality and severe morbidity among children in the Kenyan Coast. Trop Med Int Health 1996;1:139-46.
9. Binka FN, Kubaje A, Adjuik M, Williams LA, Lengeler C, Maude CH, et al. Impact of Permethrine impregnated bednets on child mortality in Kassena-Nankana district of Ghana: a randomized controlled trial. Trop Med Int Health 1996;1:147-54.
10. Lengeler C, Cattani J, de Savigny D, editors. Net gain: a new method for preventing malaria deaths. Ottawa, Canada: International Development Centre/World Health Organization; 1996.
11. World Health Organization. Toward healthy women counseling guide: ideas from the gender and health research group. Geneva: The Organization, UNDP/World Bank/WHO Special Programme for Research and Training in Tropical Diseases (TDR). Report No.: TDR/GEN/95.1
12. World Health Organization. Tropical diseases research: progress 1995-96. 13th Programme Report. Geneva: The Organization, UNDP/World Bank/WHO Special Programme for Research and Training in Tropical Diseases(TDR), Geneva.
13. Pagnoni F, Convelbo N, Tiendrebeago H, Cousens S, Esposito F. A community-based programme to provide prompt and adequate treatment of presumptive malaria in children. Trans R Soc Trop Med Hyg 1997;91:512-7.
14. Carlson J, Olson K, Higgs S, Beaty B. Molecular genetic manipulation of mosquito vectors. Annu Rev Entomol 1995;40:359-88.
15. Omumbo J, Ouma J, Rapouda B, Craig M, le Sueur D, Snow RW. Mapping malaria transmission intensity using geographic information systems: an example from Kenya. Ann Trop Med Parasitol. In press 1998.
16. Mons B, Klasen E, van Kessel R, Nchinda T. Partnerships between South and North crystallizes around malaria. Science 1998;279:498-9.
